# T cell receptor repertoire among women who cleared and failed to clear cervical human papillomavirus infection: An exploratory proof-of-principle study

**DOI:** 10.1371/journal.pone.0178167

**Published:** 2018-01-31

**Authors:** Krystle A. Lang Kuhs, Shih-Wen Lin, Xing Hua, Mark Schiffman, Robert D. Burk, Ana Cecilia Rodriguez, Rolando Herrero, Christian C. Abnet, Neal D. Freedman, Ligia A. Pinto, David Hamm, Harlan Robins, Allan Hildesheim, Jianxin Shi, Mahboobeh Safaeian

**Affiliations:** 1 National Cancer Institute, NIH, Bethesda, Maryland, United States of America; 2 Vanderbilt University Medical Center, Nashville, Tennessee, United States of America; 3 Albert Einstein College of Medicine, Bronx, New York, United States of America; 4 Proyecto Epidemiológico Guanacaste, Fundación INCIENSA, San José, Costa Rica; 5 Prevention and Implementation Group, International Agency for Research on Cancer, Lyon, France; 6 HPV Immunology Laboratory, Leidos Biomedical Research, Inc., Frederick National Laboratory for Cancer Research, Frederick, Maryland, United States of America; 7 Adaptive Biotechnologies, Seattle, Washington, United States of America; 8 Fred Hutchinson Cancer Research Cancer, Seattle, Washington, United States of America; Fondazione IRCCS Istituto Nazionale dei Tumori, ITALY

## Abstract

**Background:**

It is unknown why a minority of women fail to clear human papillomavirus (HPV) and develop precancer/cancer. Differences in T-cell receptor (TCR) repertoires may identify HPV16-infected women at highest-risk for progression to cancer. We conducted a proof-of-principle study nested within the Guanacaste HPV Natural History Study to evaluate the utility of next-generation sequencing for interrogating the TCR repertoires among women who cleared and failed to clear cervical HPV16.

**Methods:**

TCR repertoires of women with HPV16-related intraepithelial neoplasia grade 3 or higher (CIN3+; n = 25) were compared to women who cleared an incident HPV16 infection without developing precancer/cancer (n = 25). TCR diversity (richness and evenness) and relative abundance (RA) of gene segment (V [n = 51], D [n = 2], J [n = 13]) usage was evaluated; receiver operating curve analysis assessed the ability to differentiate case-control status.

**Results:**

TCR repertoire richness was associated with CIN3+ status (*P* = 0.001). Relative abundance (RA) of V-gene segments was enriched for associations between cases and controls. A single V-gene (*TRBV6-7)* was significantly associated with CIN3+ status (RA = 0.11%, 0.16%, among cases and controls, respectively, Bonferroni *P* = 0.0008). The estimated area under the curve using richness and V-gene segment RA was 0.83 (95% confidence interval: 0.73–0.90).

**Conclusions:**

Substantial differences in TCR repertoire among women with CIN3+ compared to women who cleared infection were observed.

**Impact:**

This is the first study to use next-generation sequencing to investigate TCR repertoire in the context of HPV infection. These findings suggest that women with HPV16-associated cervical lesions have significantly different TCR repertoires from disease-free women who cleared HPV16 infection.

## Introduction

Persistent human papillomavirus (HPV) infection is a necessary cause of cervical cancers [1, 2], with high-risk HPV types 16 and 18 together accounting for approximately 70% of all cases [3]. While approximately 90% of women will contract a cervical HPV infection during their lifetime [4], only a small percentage will progress to precancer/cancer [5, 6]. It is currently unknown why only a minority of women develops HPV-associated precancerous lesions, while the majority of HPV infected women are able to clear the virus.

Although the exact mechanisms are unknown, numerous studies have shown that T-cells as well as genetic factors that mediate T-cell responses are integral for controlling HPV infection [7–10]. T-cells are able to respond to infection by using their T-cell receptors (TCR) to bind and recognize foreign antigens presented by major histocompatibility complex (MHC) molecules located on the surface of infected cells. The specificity of T-cells for an individual antigenic peptide-MHC complex is primarily determined by the amino-acid sequence in the hypervariable complementarity-determining region 3 (CDR3) of the α- and β-chains of the TCR [11]. During T-cell development, each individual T-cell generates a unique TCR sequence through a process called somatic DNA recombination, where numerous noncontiguous variable (V), diversity (D), and joining (J) gene segments encoded within the germline are rearranged to form a unique TCR sequence within an immature T-cell. During this process, nucleotides can also be randomly inserted and/or deleted between the VDJ gene segments, further adding to the combinatorial diversity of the TCR. Thus, the rearrangement of multiple V, D and J gene segments as well as the random insertion and/or deletion of nucleotides at the gene junctions can theoretically result in up to 1x10^18^ unique CDR3 sequences [12, 13]. It is generally believed that a diverse TCR repertoire is necessary for mounting an effective adaptive immune response against the immense variety of existing human pathogens [11].

Thus, differences in TCR repertoires between individuals may be a contributing factor in the development of cervical lesions. However, characterization of TCR repertoires among HPV-infected women has yet to be conducted. Historically, detailed characterization of the TCR repertoire has been hindered by traditional sequencing techniques which are unable to efficiently interrogate the short (15–60 nucleotides), highly diverse regions within each TCR sequence. An emerging field of immunosequencing has recently been developed allowing for the interrogation of the TCR repertoire at a level of detail impossible just a few years ago [14]. Yet, the utility of this technology for detecting differences in TCR repertoires between women who cleared a cervical HPV infection compared to women who developed precancer/cancer is currently unknown.

Therefore, prior to evaluating whether differences in TCR repertoires are predictive of HPV persistence, we first designed a proof-of-principle study nested within the Guanacaste HPV Natural History Study. The goal of this study was to evaluate the utility of this technology for detecting TCR repertoire differences in the context of cervical HPV16 infection. To maximize the chances of detecting potential differences, we compared the TCR β-chain CDR3 repertoires of women at the extremes of the HPV infection continuum: i) women with histologically confirmed HPV16-related CIN3+ (persistently infected with HPV16, n = 25) and ii) women who cleared an incident HPV16 infection within 12 months and did not develop precancer/cancer over the course of follow-up (n = 25).

## Materials and methods

### Study cohort

This study was nested within the Guanacaste HPV Natural History Study (NHS), a well-characterized, longitudinal cohort study conducted in Guanacaste, Costa Rica. The study design and methods have been previously described elsewhere [15, 16]. Briefly, the NHS is a population-based cohort of 10,049 women aged 18 and older recruited from Guanacaste, Costa Rica between June 1993 and December 1994; the follow-up phase of the study lasted 7 years (1994–2001). The main objective of the NHS was to prospectively examine the natural history of HPV infection and precancer (CIN2-3).

At enrollment and at each follow-up visit, sexually-active women underwent a pelvic exam and blood specimen collection. During the pelvic exam, exfoliated cervical cells were collected for conventional and liquid-based cytology (ThinPrep; Hologic Corporation) using a Cervex broom-type brush (Unimar); a second sample was collected with Dacron swabs for HPV DNA detection and genotyping. The frequency of follow-up visits varied by the cytologic findings of the preceding visit. Six month follow-up visits were conducted among women with evidence of low-grade squamous intraepithelial (LSIL); annual follow-up visits were scheduled for women who had atypical squamous cells of undetermined significance (ASCUS), were HPV positive or reported 5 or more sexual partners. Annual follow-up visits were also conducted for a random subset of cytologically normal HPV-negative women as well as a subset of virgins. The remaining cytologically normal HPV-negative women were referred to the Costa Rican medical system for continued routine screening and were seen once more by study clinicians either at 5, 6 or 7 years post-enrollment. Women with high grade cytology were referred for colposcopic evaluation and were censored from further screening. The study protocol was reviewed and approved by the National Cancer Institute (NCI) and a Costa Rica Institutional Review Board (IRB). For this specific study, the NCI IRB waived the need for consent, and the data was accessed anonymously.

### Case control definitions

We selected a total of 50 participants (25 cases and 25 controls) singly infected with an incident cervical HPV16 infection; cases of prevalent HPV16 infections were excluded. Given that this study was initially designed as an exploratory proof-of-concept study, in order to maximize the chances of detecting differences, we selected cases and controls at the extremes of the cancer continuum. Cases were defined as participants with histologically confirmed HPV16-related CIN grade 3 or higher (CIN3+; included 2 cases of invasive cancer); CIN3+ was attributed to the HPV type detected by PCR in the directly preceding cervical cytology specimen that led to colposcopy referral for the CIN3+ lesion. Controls were defined as participants without evidence of precancer or cancer, but who had had a single incident HPV16 infection that cleared within 12 months. For cases, blood samples from the visit when the lesion was first identified were used (median 149 days between lesion detection and blood sampling, interquartile range of 90 to 200 days); for controls, blood samples from the visit when the infection was no longer detected were used. Cases and controls were matched on age (± 5 years) and year of sample collection.

### HPV DNA testing

DNA extracted from exfoliated cervical cell specimens was amplified using the MY09/MY11 L1 degenerate primer polymerase chain reaction (PCR) method with AmpliTaq Gold polymerase (TaqGold; Perkin-Elmer-Cetus) [17]. Following amplification, dot-blot hybridization was used for HPV genotyping [18].

### Blood collection and separation/freezing of buffy coat

Using standard procedures, 15mL of blood were collected in heparinized tubes. Tubes were kept at 1–4°C until centrifuged at 900G for 20 minutes and aliquotted into separate 2mL vials of plasma, buffy coat and red blood cells. Aliquots were initially frozen at -30°C and then stored at -80°C.

### Genomic DNA extraction, amplification, sequencing and bioinformatic analysis

QIAGEN kits (Qiagen, Venlo, Limburg) were used to extract genomic DNA from buffy coat specimens. The TCR-β CD3 region (consisting of recombined V, D and J gene regions) was amplified and sequenced from an average of 1200ng DNA per sample using the ImmunoSEQ assay (Adaptive Biotechnologies, Seattle, WA). In this assay, a multiplex PCR system was used to amplify CDR3β sequences from sample DNA. The 60-base-pair fragment is sufficient to identify the VDJ region spanning each unique CDR3β. Amplicons were sequenced using the Illumina platform. TCR-β V, D and J gene definitions were provided by the IMGT database (www.imgt.org). The assay is quantitative, having used a complete synthetic repertoire of TCRs to establish an amplification baseline and adjust the assay chemistry to correct for primer bias. The resulting data is filtered and clustered using both the relative frequency ratio between similar clones and a modified nearest-neighbor algorithm, to merge closely related sequences and remove both PCR and sequencing errors. Data was analyzed using the ImmunoSEQ analyzer toolset. For quality controls, 5 blinded duplicates from each group were inserted for a total of 60 samples; 50 patients and 10 replicates.

### Statistical analyses

#### Relative abundance

We calculated the relative abundances (RAs) of each V (n = 51), D (n = 2) and J (n = 13) gene individually as well as RAs of each unique VDJ combination (51*2*13 = 1326 possible VDJ combinations) and tested for associations with case-control status using the Wilcoxon rank sum test. We identified significant associations using a false discovery rate (FDR) correction [19].

#### TCR diversity

TCR repertoire diversity is reflected by both the richness and evenness of the TCR amino acid sequences. Richness is defined as the total number of unique TCR amino acid sequences, while evenness is a measure of how evenly distributed the RAs of each unique TCR amino acid sequence are within a particular repertoire. For this analysis evenness was quantified using Pielou’s Evenness, $[-\sum_{i=1}^{n}p_{i}log(p_{i})]/log(n)$, assuming *n* unique TCR amino acid sequences with RAs denoted as (*p*_1_,⋯,*p_n_*). Both richness and evenness estimates are heavily dependent on the total number of sequence reads per sample; thus, we performed rarefaction, an analytic technique frequently used in microbiomics [20], to eliminate the confounding effect of varying numbers of sequences. Briefly, for a given rarefaction depth (*M*), we randomly selected *M* sequences without replacement for each sample with more than *M* sequences and calculated both TCR richness and evenness; samples with less than *M* sequences were excluded from analysis. We repeated the rarefaction 20 times and estimated the average richness and evenness. For each rarefaction depth, we used a t-statistic to test for significant differences in richness or evenness between cases and controls.

#### Assay reproducibility

To investigate data reproducibility, intraclass correlation coefficients (ICCs) for i) TCR richness (number of unique TCR amino acid sequences) and ii) relative abundances (RAs) of V, D and J genes were calculated based on blind duplicated samples. The ICC for TCR richness was 95.6%. DNA sequencing identified 51 different V genes, 2 different D genes and 13 different J genes; for the 66 genes sequenced, the average ICC was 76.4%, 26 had ICCs greater than 90% and 42 had ICCs greater than 80%.

#### ROC curve analysis

We performed receiver operating curve (ROC) analysis to evaluate the utility of V gene usage and TCR richness to differentiate between cases and controls. In each analysis, we used the random forest algorithm to build classifiers. The area under the curve (AUC) and its 95% confidence interval were calculated by cross-validation coupled with resampling without replacement. In each resampling, we randomly partitioned 49 samples into six groups (five groups with 4 cases and 4 controls and one group with 5 cases and 4 controls) and derived six AUC values using cross-validations with the specified discovery-validation assignment. We repeated this process 100 times by randomly assigning group membership to eliminate the variation due to group assignment when sample size was small. The final AUC was estimated as the average of the 600 AUC values and the 95% confidence interval was estimated as the 2.5% and 97.5% quantiles.

Following Holmes et al. [21], we used random forest [22] together with the leave-one-out (LOO) procedure to construct ROC curves. In each iteration of LOO, we took out one sample, built a random forest classifier using the rest of the samples, and calculated the predicted probability for the sample as a case using the trained classifier. We plotted the ROC curve using the predicted probabilities for all samples based on the LOO procedure and calculated AUC using an R package ROCR.

## Results

### Participant characteristics

Compared to controls, cases were younger (median age 34 versus 38), had a lower lifetime number of sexual partners (median 1 versus 2) and more likely to be never smokers (92% versus 88%), however, none of these differences were statistically significant (*P*≥0.08) (S1 Table).

### Association of TCR repertoire diversity and CIN3+ development

We assessed the diversity of the TCR repertoire by case-control status using two different diversity metrics; i) richness, defined as the total number of unique TCR amino acid sequences contained within each TCR repertoire and ii) evenness, a measure of how evenly distributed the relative abundances of each unique TCR amino acid sequence are within a particular repertoire.

**Fig 1:**
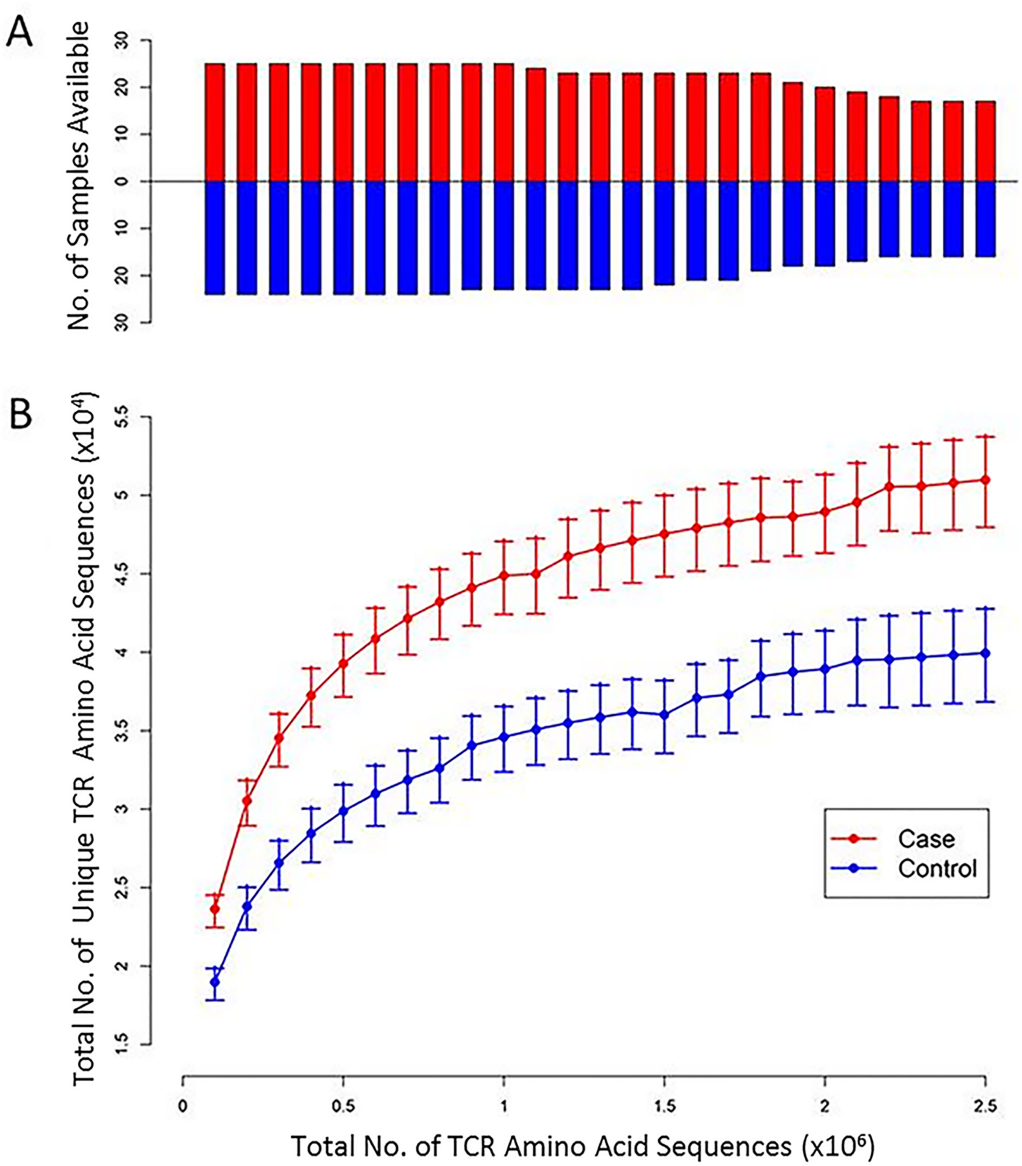
T cell repertoire richness (defined as the total number of unique CDR3 amino acid sequences within the TCR β-chain) was greatest among women with HPV16-related CIN3+ (cases) compared to women who cleared an incident cervical HPV16 infection without precancer/cancer development (controls); findings were independent of TCR sequencing depth. TCR repertoire richness (defined as the total number of unique CDR3 amino acid sequences within the TCR β-chain) was calculated by rarefying to different sequence read depths. Given that the TCR diversity estimate is heavily dependent on the total number of sequences per sample, we performed rarefaction to eliminate the confounding effect of varying numbers of sequences. Panel A shows the number of cases (red) and controls (blue) available for calculating TCR diversity at each read depth (range 100,000 reads [first box] to 3,000,000 [last box]); each graphical box represents a 120,000 increase in read depth. Panel B depicts the average TCR richness calculated for each read depth shown in Panel A separately for cases (red) and controls (blue). Bars represent the standard deviation of the estimated richness in cases and in controls.

Irrespective of sequencing depth, TCR repertoire richness was significantly higher among women with HPV16-related CIN3+ compared to women who had cleared an incident cervical HPV16 infection (*P* = 0.001, smallest p-value), Fig 1; however, no significant differences in TCR repertoire evenness was observed by case-control status (*P* = 0.74, smallest p-value), S1 Fig. Adjusting for age, smoking status and the number of sexual partners did not change the results in respect to either richness or evenness.

### Association of VDJ gene usage within the TCR CDR3 β-chain and CIN3+ development

The amino acid sequence of each TCR (the totality of which comprise the overall diversity of the TCR repertoire) is largely determined by the usage of differing combinations of individual V (n = 51), D (n = 2) and J gene segments (n = 13) [11]. Given the increased TCR repertoire richness among CIN3+ cases, we assessed whether RAs of any particular VDJ gene recombinations were associated with case-control status. After multiple testing correction, we did not detect significant associations between the RA of any particular VDJ gene recombinations and case-control status (S2 Table). However, the quantile-quantile (Q-Q) plot of *P* values strongly deviated from the expected uniform distribution under the global null hypothesis, suggesting that many VDJ gene recombinations were modestly associated with case-control status (S2 Fig).

We next examined the RAs of each V, D and J gene segment individually. A deviation from the null distribution was observed only in the Q-Q plot for the V gene segments, suggesting that the overall association was primarily driven by the V gene segments (S3 Fig). Of the V gene segments assessed (S3 Table), RAs of the V gene, *TRBV6-7*, were significantly associated with case-control status (nominal P = 1.2×10^−5^ and Bonferroni corrected P = 0.0008). The average RA of *TRBV6-7* was 0.11% (95% CI: 0.07%-0.15%) and 0.16% (95% CI: 0.13%-0.19%) among cases and controls, respectively (S4 Fig). RAs of *TRBV6-7* were highly reproducible with an ICC of 0.81.

### Differentiation of case-control status

**Fig 2:**
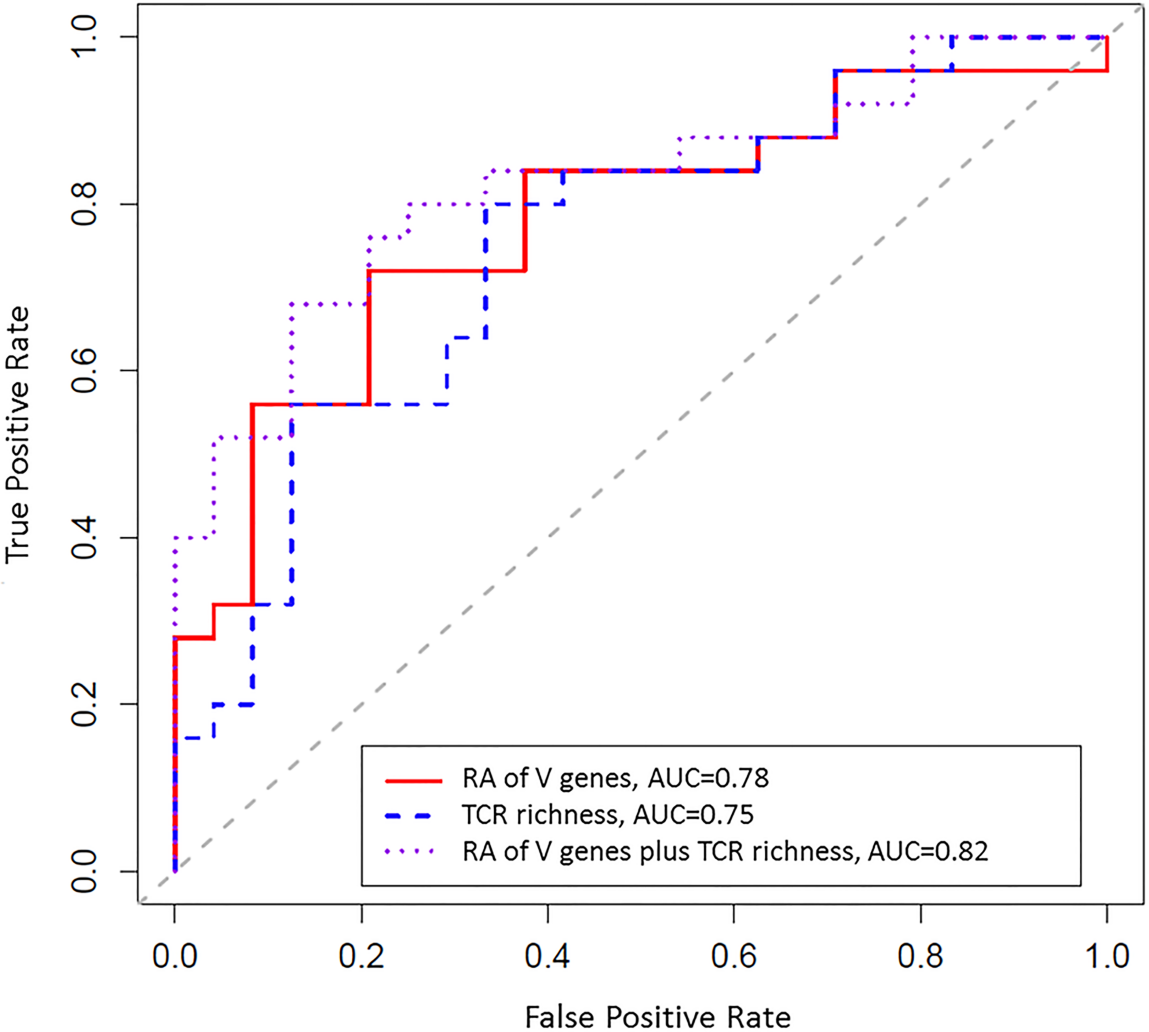
TCR diversity and relative abundance of the V gene usage within the TCR together strongly differentiated women with HPV16-related CIN3+ (cases) from women who cleared an incident cervical HPV16 infection without precancer/cancer development (controls). Receiver operating curves (ROC) were generated using random forest together with the leave-one-out (LOO) procedure for i) relative abundances (RA) of V genes (red line); ii) TCR repertoire richness (dotted blue line); and iii) RA of V genes and TCR repertoire richness combined (dotted purple line). Reported AUCs and their confidence intervals were based on resampling without replacement.

We performed AUC analysis to evaluate utility of TCR repertoire diversity (assessed by TCR richness), and RA of V gene usage within the CDR3 β-chain of the TCRs to differentiate cases from controls. The AUC and its confidence interval (CI) were estimated using random forest [22] and resampling without replacement. The estimated AUC was 0.75 (95% CI: 0.60–0.88) using only the TCR richness; 0.79 (95% CI: 0.69–0.86) using RAs of V genes alone; and was increased to 0.83 (95% CI: 0.73–0.90) when incorporating both TCR richness and V gene RAs. The ROC curve presented in Fig 2 was based on random forest coupled with leave-one-out procedure following Holmes [21], which gave similar estimates of AUC values: 0.78 using V gene RAs alone and 0.82 using both richness and V gene RAs.

## Discussion

In this exploratory case-control study nested within the Guanacaste HPV Natural History Study, using a novel deep-sequencing approach to interrogate the CDR3 β-chain region of the TCR, we were able to detect differences in the TCR repertoires of women who cleared and failed to clear their HPV16 infection. We found that women who developed HPV16-associated CIN3+ (cases) had a more diverse T-cell repertoire (i.e. more unique TCR sequences [TCR richness]) at the time of lesion diagnosis compared to women with an incidently detected cervical HPV16 infection that cleared within 12 months (controls). Further, we found that the relative abundance (RA) of a specific V gene (*TRBV6-7*) was on average twice as abundant among controls compared to cases. The combination of both TCR richness and V gene usage was strongly predictive of case-control status (AUC = 0.83). To our knowledge, this is the first study to investigate the association of the TCR repertoire with HPV16-related CIN3+.

The specificity of T-cells for an individual antigenic peptide-MHC complex is primarily determined by the CDR3 β-chains of the TCR; thus, sequencing of this region provides a measure of the overall diversity of the T-cell repertoire [11]. We found that women who developed HPV16-associated CIN3+ had a more diverse TCR repertoire as defined by TCR richness; however, no significant differences were observed with respect to TCR evenness. A possible explanation for this finding may be the timing of the blood collection. For cases, the blood sample tested was collected at the time of diagnosis of the lesion compared to controls whose blood samples tested were at the time that infection had resolved. Although unlikely given that HPV causes a local rather than systemic infection, cases may have had a higher T-cell density within the circulating blood (given that they may have been actively fighting the infection). This could have resulted in more T-cells being sequenced per milliliter of blood for cases compared to controls even though the same amount of blood was collected from all participants. The greater the number of T-cells sequenced, the higher the likelihood of identifying unique and rare TCR sequences, thus resulting in a greater estimate of TCR richness. While the same amount of DNA was sequenced for each sample, we did not have information regarding the number of T-cells in each sample and therefore, could not control for T-cell number in our analysis. Following the same logic, we would have hypothesized that HPV-specific T-cells would make up a larger proportion of the TCR repertoire among cases compared to controls, thus skewing the distribution of TCR sequence relative abundances resulting in a lower estimate of TCR repertoire evenness among cases. Contrary to our hypothesis, no significant differences were observed with respect to TCR repertoire evenness. A possible explanation for this finding may be that, among cases, the HPV-specific T-cells may have been localized to the cervix (the site of HPV-infection) and not circulating within the peripheral blood; thus artificially resulting in a greater estimate of evenness. In line with this reasoning, previous studies have demonstrated that a large pool of polyclonal HPV-specific T-cells localize to the genital tract among individuals with cervical cancer [23]; however, in this current study we did not sequence T-cells from the cervix.

We did not detect significant associations between the RAs of individual VDJ recombinants and case-control status, although interestingly the Q-Q plot of *P* values strongly suggested that many VDJ recombinants were modestly associated with case-control status, even in this small study. Thus, larger sample size has the potential to identify multiple significant associations. We detected a single V gene, *TRBV6-7*, whose RAs were significantly associated with case-control status. Preferential expansion of T-cells with certain TCR V genes has been commonly observed during HIV, Epstein-Barr virus (EBV) and cytomegalovirus infection in humans [11]. To our knowledge, no study to date has reported associations between TCR V genes and HPV infection. It is unclear what role, if any, *TRBV6-7* plays in control of HPV infection given that for this study, the entire TCR repertoire was sampled and sequenced.

A limitation of this study is that we did not select for effector or memory T-cell subsets nor did we isolate HPV-specific T-cells; thus, we cannot make conclusions regarding the diversity of the HPV-specific T-cell response or the preferential selection of specific V, D, J gene sequences (such as *TRBV6-7*). Furthermore, given the cross-sectional study design, we are unable to determine whether the associations observed were present before infection or whether they were the consequence of infection. Thus, our findings should be interpreted as reflecting the state of the immune system during infection/persistence or post-infection clearance and may not necessarily be predictive of who will clear HPV infection. We note that the median age of controls was 34 years; thus they could have had HPV infection prior to enrollment in the Guanacaste Natural History Study, which may have affected the associations observed in our current analysis. Finally, while it is generally accepted that HLA type is an important risk factor for developing cervical cancer, we had HLA typing results for only a limited number of women and could not exclude HLA type as a potential confounder.

A major strength of this study was that it is the first to investigate TCR diversity in the context of HPV infection using cutting edge next-generation high-throughput sequencing technique. This study provides proof-of-concept that next-generation high-throughput sequencing can be effectively used to interrogate the T-cell repertoire of women with and without HPV-related precancer; a feat that until recently had been impossible with traditional sequencing methods. Furthermore, we used samples from a large, well-characterized longitudinal cohort study that closely followed participants for the presence of precancer/cancer and therefore permitted the identification of cases who developed disease after an incident HPV infection and controls who cleared incident infections. Additionally, the NHS collected approximately 7 years of follow-up, which has the potential to provide numerous time points for future expansion of this study.

In conclusion, in the first proof-of-concept study using next-generation sequencing to investigate TCR repertoire in the context of HPV infection, our findings suggest that women with active HPV infection and cervical lesions have significantly different TCR repertoires from disease-free women who had cleared their infections. Larger prospective studies testing samples both before and during HPV infection are needed to replicate these early findings and to further understand the role of T-cell repertoires in the etiology of cervical precancer/cancer.

## Supporting information

S1 Table Participant characteristics. ^1^Cases were defined as participants with histologically confirmed HPV16-related CIN grade 3 or higher (CIN3+). Controls were defined as participants without evidence of precancer or cancer, but who had had a single incident HPV16 infection that cleared within 12 months. For cases, blood samples from the visit when the lesion was first identified were used; for controls, blood samples from the visit when the infection was no longer detected were used. Cases and controls were matched on age (± 5 years) and year of sample collection.
(DOC)

S2 Table Relative abundance (RA) of each VDJ gene recombination. (XLS)

S3 Table Mean relative abundance (RA) of each individual V, D, and J gene. (XLS)

S1 Fig T cell repertoire evenness (as defined by Pielou’s Evenness) did not differ significantly between women with HPV16-related CIN3+ (cases) and women who cleared an incident cervical HPV16 infection without precancer/cancer development (controls); findings were independent of TCR sequencing depth. TCR repertoire evenness (defined by the Pielou’s Evenness [higher number indicates a greater diversity]) was calculated by rarefying to different sequence read depths. Panel A shows the number of cases (red) and controls (blue) available for calculating TCR diversity at each read depth (range 100,000 reads [first box] to 3,000,000 [last box]); each graphical box represents a 120,000 increase in read depth. Panel B depicts the TCR diversity calculated for each read depth shown in Panel A separately for cases (red) and controls (blue). Bars represent the standard deviation of the estimated evenness in cases and in controls.
(DOC)

S2 Fig Quantile-quantile (Q-Q) plot of *P* values produced by the Wilcoxon Signed-rank test. Wilcoxon Signed-rank test for the association between VDJ gene recombinations and case-control status.
(DOC)

S3 Fig Quantile-quantile (Q-Q) plot of *P* values generated using the Wilcoxon Signed-rank test. Wilcoxon Signed-rank test for the association between: a) V gene; b) J gene; and c) DJ gene segments and case-control status. Of note, given that the D gene has only 2 variants, the D gene segments are shown combined with the J gene segments in Panel C for presentation purposes.
(DOC)

S4 Fig Mean relative abundance of the *TRBV6-7* gene segment by case-control status. (DOC)
