# Supplementary material for: T cell receptor repertoire among women who cleared and failed to clear cervical human papillomavirus infection: An exploratory proof-of-principle study
Source: PLoS One. 2018 Jan 31;13(1):e0178167. doi: 10.1371/journal.pone.0178167 (PMC5791954; doi:10.1371/journal.pone.0178167)
Supplement: S1 Table — 1Cases were defined as participants with histologically confirmed HPV16-related CIN grade 3 or higher (CIN3+). Controls were defined as participants without evidence of precancer or cancer, but who had had a single incident HPV16 infection that cleared within 12 months. For cases, blood samples from the visit when the lesion was first identified were used; for controls, blood samples from the visit when the infection was no longer detected were used. Cases and controls were matched on age (± 5 years) and year of sample collection. (DOC) [file pone.0178167.s001.doc]

| **Supplemental Table S1**: Participant characteristics. | | |
| --- | --- | --- |
|  |  |  |
|  | **Case**^1^ (N=25) | **Control**^1^ (N=25) |
| **Age** |  |  |
| Median (IQR) | 34 (27-47) | 38 (30-49) |
| Range | 22-64 | 22-66 |
| **Age at First Sex** |  |  |
| Median (IQR) | 17 (16-19) | 18 (16-19) |
| **Number of Sexual Partners** |  |  |
| Median (IQR) | 1 (1-3) | 2 (1-4) |
| **Smoking Status**, N(%) |  |  |
| Never | 23 (92.0) | 22 (88.0) |
| Current | 0 (0.0) | 1 (4.0) |
| Former | 2 (8.0) | 2 (8.0) |
|  |  |  |
| ^1^Cases were defined as participants with histologically confirmed HPV16-related CIN grade 3 or higher (CIN3+). Controls were defined as participants without evidence of precancer or cancer, but who had had a single incident HPV16 infection that cleared within 12 months. For cases, blood samples from the visit when the lesion was first identified were used; for controls, blood samples from the visit when the infection was no longer detected were used. Cases and controls were matched on age (± 5 years) and year of sample collection. | | |
