# Supplementary material for: T cell receptor repertoire among women who cleared and failed to clear cervical human papillomavirus infection: An exploratory proof-of-principle study
Source: PLoS One. 2018 Jan 31;13(1):e0178167. doi: 10.1371/journal.pone.0178167 (PMC5791954; doi:10.1371/journal.pone.0178167)
Supplement: S1 Fig — TCR repertoire evenness (defined by the Pielou’s Evenness [higher number indicates a greater diversity]) was calculated by rarefying to different sequence read depths. Panel A shows the number of cases (red) and controls (blue) available for calculating TCR diversity at each read depth (range 100,000 reads [first box] to 3,000,000 [last box]); each graphical box represents a 120,000 increase in read depth. Panel B depicts the TCR diversity calculated for each read depth shown in Panel A separately for cases (red) and controls (blue). Bars represent the standard deviation of the estimated evenness in cases and in controls. (DOC) [file pone.0178167.s004.doc]

**Supplemental Fig. S1**: T cell repertoire evenness (as defined by Pielou’s Evenness) did not differ significantly between women with HPV16-related CIN3+ (cases) and women who cleared an incident cervical HPV16 infection without precancer/cancer development (controls); findings were independent of TCR sequencing depth.


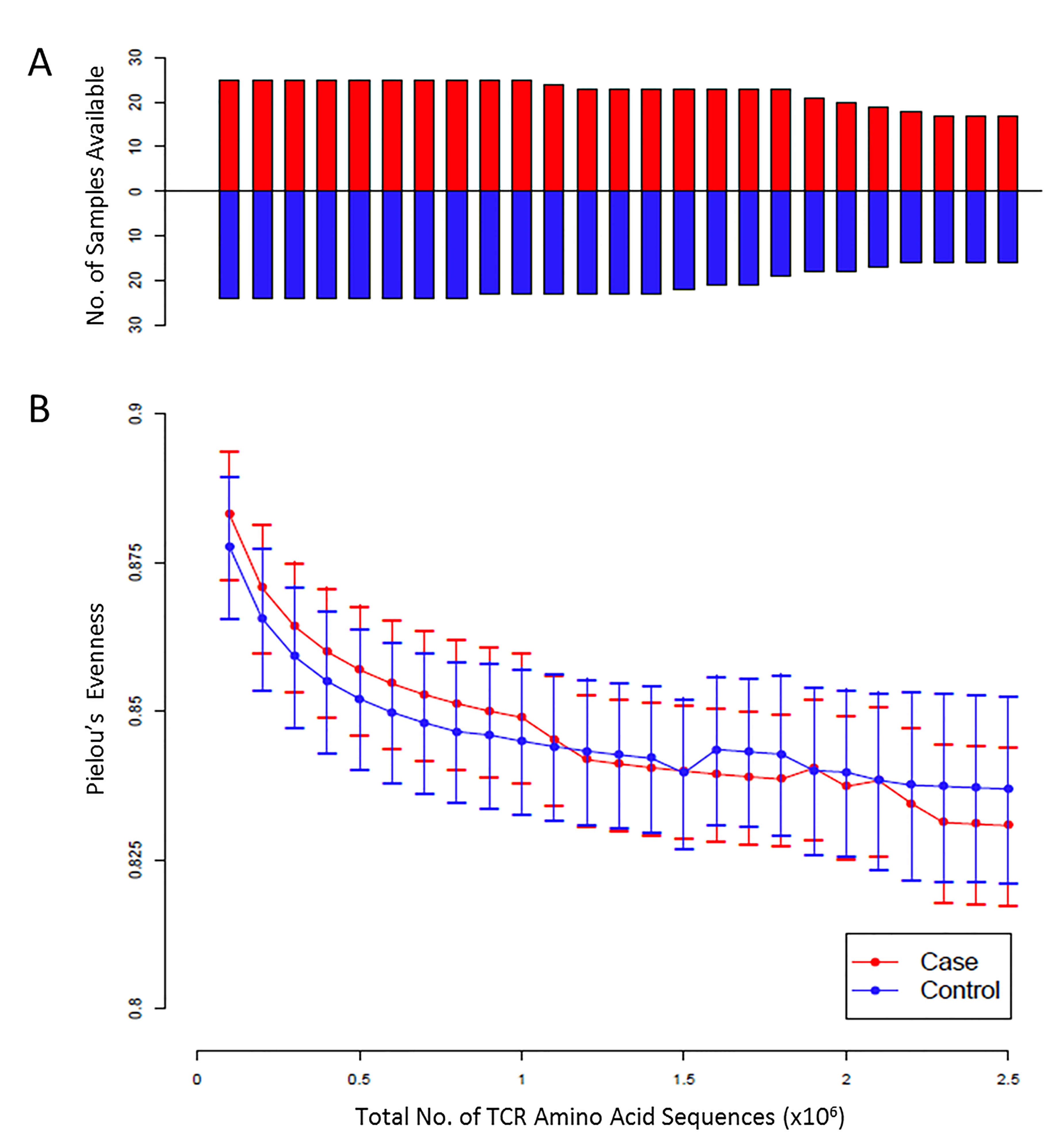


TCR repertoire evenness (defined by the Pielou’s Evenness [higher number indicates a greater diversity]) was calculated by rarefying to different sequence read depths. Panel A shows the number of cases (red) and controls (blue) available for calculating TCR diversity at each read depth (range 100,000 reads [first box] to 3,000,000 [last box]); each graphical box represents a 120,000 increase in read depth. Panel B depicts the TCR diversity calculated for each read depth shown in Panel A separately for cases (red) and controls (blue). Bars represent the standard deviation of the estimated evenness in cases and in controls.
