# Supplementary material for: T cell receptor repertoire among women who cleared and failed to clear cervical human papillomavirus infection: An exploratory proof-of-principle study
Source: PLoS One. 2018 Jan 31;13(1):e0178167. doi: 10.1371/journal.pone.0178167 (PMC5791954; doi:10.1371/journal.pone.0178167)
Supplement: S3 Fig — Wilcoxon Signed-rank test for the association between: a) V gene; b) J gene; and c) DJ gene segments and case-control status. Of note, given that the D gene has only 2 variants, the D gene segments are shown combined with the J gene segments in Panel C for presentation purposes. (DOC) [file pone.0178167.s006.doc]

**Supplemental Fig. S3**: Quantile-quantile (Q-Q) plot of *P* values generated using the Wilcoxon Signed-rank test.


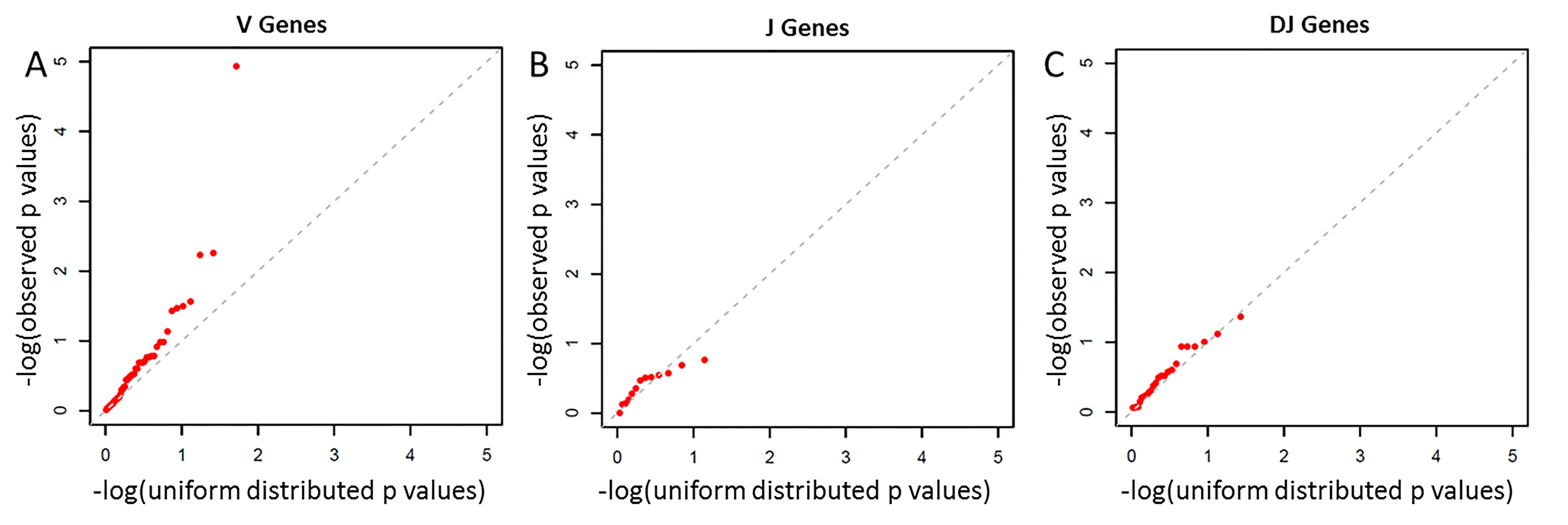


Wilcoxon Signed-rank test for the association between: a) V gene; b) J gene; and c) DJ gene segments and case-control status. Of note, given that the D gene has only 2 variants, the D gene segments are shown combined with the J gene segments in Panel C for presentation purposes.
