# Supplementary material for: T cell receptor repertoire among women who cleared and failed to clear cervical human papillomavirus infection: An exploratory proof-of-principle study
Source: PLoS One. 2018 Jan 31;13(1):e0178167. doi: 10.1371/journal.pone.0178167 (PMC5791954; doi:10.1371/journal.pone.0178167)
Supplement: S4 Fig — (DOC) [file pone.0178167.s007.doc]

**Supplemental Fig. S4**: Relative abundance of the *TRBV6-7* gene segment by case-control status.
